# Current status and research hotspots of pediatric nephrotic syndrome: a bibliometric analysis (2011–2025)

**DOI:** 10.3389/fped.2026.1870546

**Published:** 2026-07-09

**Authors:** Wenyin Yang, Yu Pei, Chaoyang Wang, Mingxia Tang, Jiadong Xie, Jing Zhao

**Affiliations:** 1 Affiliated Hospital of Nanjing University of Chinese Medicine, Nanjing, China; 2 School of Artificial Intelligence and Information Technology, Nanjing University of Chinese Medicine, Nanjing, China

**Keywords:** CiteSpace, pediatric kidney disease, pediatric nephrotic syndrome, visual analysis, VOSviewer

## Abstract

**Objectives:**

The study aimed to conduct a bibliometric analysis of pediatric nephrotic syndrome (NS) and indicate its current status, hot spots, and directions of future studies.

**Methods:**

The literature data were collected from the Web of Science Core Collection and Scopus. CiteSpace, VOSviewer and Biblioshiny (Bibliometrix package) were employed to conduct a visualized bibliometric analysis of pediatric NS, including countries, institutions, authors, journals, keywords, and references.

**Results:**

A total of 2,873 publications from 2011 to 2025 were included. The number of articles in this field increased in recent years. China contributed the most articles (Publications = 452), but its centrality remained at 0.01. The institution with the most publications was Assistance Publique Hopitaux Paris in France (Publications = 77). Iijima Kazumoto ranked first with 58 publications. The keyword analysis revealed “nephrotic syndrome,” “children,” and “focal segmental glomerulosclerosis” as the most prominent keywords. Clusters including “nphs2,” “bone mineral density,” “glomerulonephritis,” and “periorbital edema” emerged as current popular topics. The latest burst keywords included “leukocyte count”, “lipoid nephrosis”, “unclassified drug”, “estimated glomerular filtration rate”, and “alport syndrome”.

**Conclusion:**

This study conducted a systematic bibliometric evaluation of pediatric NS, and clarified its current research status and identified future research hotspots and development trends. The exploration of novel immunosuppressants and the elucidation of complex pathogenic mechanisms remain enduring hotspots in the evolving landscape of pediatric NS.

## 1 Introduction

Nephrotic syndrome (NS) is the most common glomerular disease in pediatrics, characterized by massive proteinuria (>3.5 g/24 h), hypoalbuminemia, hyperlipidemia, and edema. The incidence varies by age, ethnicity, and region, typically ranging from 1.4 to 6.1 per 100,000 children per year (1). While the exact etiology remains partially unclear, immune dysregulation, circulating factors, and podocyte dysfunction are recognized as key mechanisms (2). Despite advancements in immunosuppressive regimens and biological agents such as Rituximab, the frequent relapses and long-term complications of treatment continue to impose a substantial burden on both patients and healthcare systems (3). Therefore, identifying emerging research trends and global collaborative frontiers is essential for optimizing personalized management strategies.

Bibliometrics is a method that applies mathematical and statistical techniques for qualitative and quantitative analysis of publications within a specific field. Unlike conventional reviews, this approach utilizes data analysis to evaluate the current research landscape, identify hot topics, and forecast the future direction of a discipline, offering a timely and intuitive means for delving into specific areas (4). Although several systematic reviews have summarized the current research status of pediatric NS, there remains a notable absence of comprehensive bibliometric analysis in this field. Hence, this study aims to review existing research and perform a systematic analysis to explore the state and trends of pediatric NS from 2011 to 2025. This study through in-depth mining of relevant literature in the Web of Science Core Collection (WoSCC) and Scopus. Using bibliometric analysis, with CiteSpace as the primary tool, VOSviewer and R4.5.2 as a supplement, this study examines publications, countries, institutions, authors, publishing journals, keywords and references within the field of pediatric NS. The goal is to summarize the current state of research and development, providing guidance for future research directions.

## 2 Materials and methods

### 2.1 Data source and search strategy

The WoS and Scopus databases are frequently used for bibliometric analysis and scientific literature visualization, as they cover nearly all of the world's most authoritative and comprehensive scientific literature. In this study, the WoSCC and Scopus databases were utilized for data collection. Additionally, to ensure retrieval accuracy, we used topic retrieval. The WoS query was formulated as follows: TS = (“Nephrotic Syndrome*”) AND TS = (Children OR Childhood OR Pediatric*). For Scopus, the search syntax was adapted to: TITLE-ABS-KEY(Nephrotic Syndrome*) AND TITLE-ABS-KEY(Children OR Childhood OR Pediatric*). The research spanned from January 1, 2011 to December 30, 2025, with the final query submission deadline set for 4 April 2026. Included publications were primarily articles and reviews, and the literature language was restricted to English. The records from WOS and Scopus were exported in Plain Text and RIS formats, respectively. This study strictly follows relevant guidelines for bibliometric reporting and research workflow transparency.

### 2.2 Data extraction and processing

**Figure 1:**
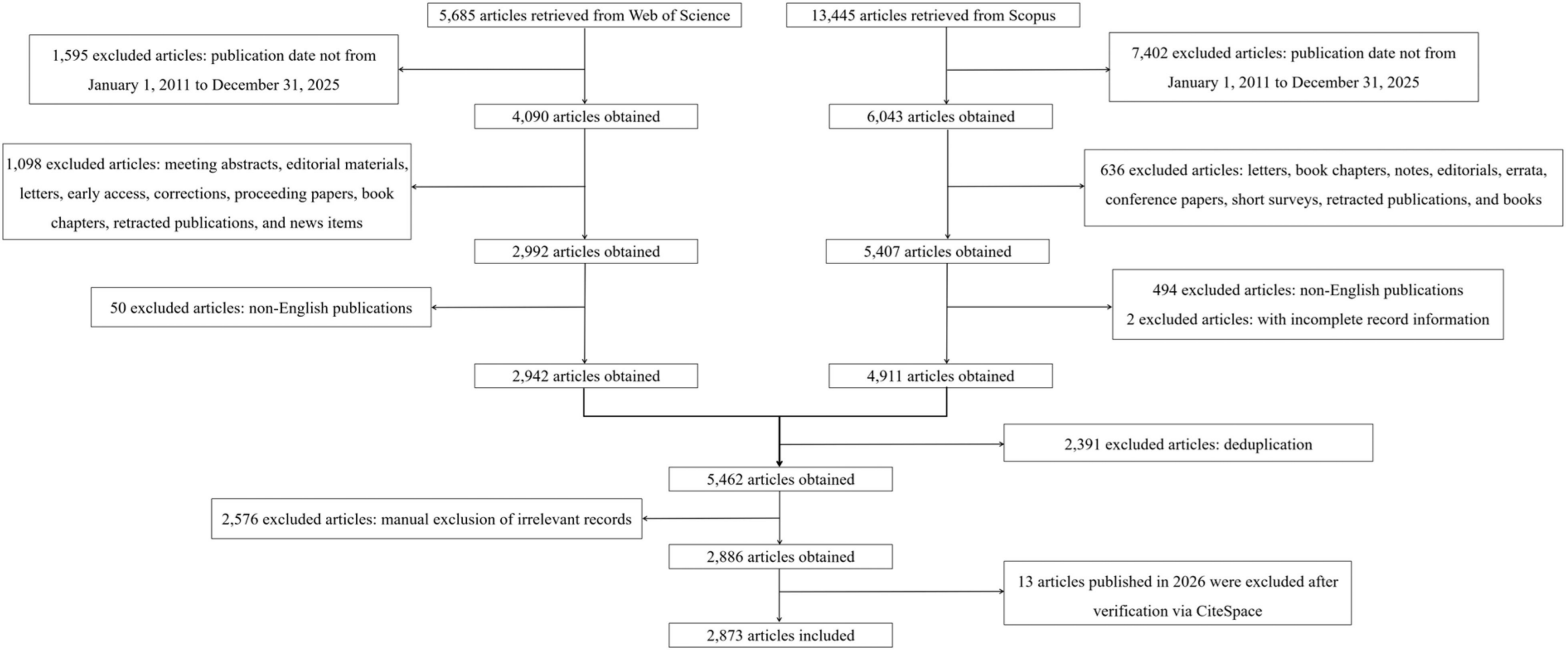
Flow chart of the study retrieval and selection process. This figure illustrates the stepwise screening process of articles retrieved from the WoS and Scopus. A total of 5,685 and 13,445 records were initially identified. After stepwise exclusion of duplicates and irrelevant studies, 2,873 articles were finally included for analysis.

The WoSCC database comprised a total of 5,685 documents, and the Scopus database contained 13,445 documents. After filtering for articles and reviews, 2,942 and 4,911 documents were finally obtained. To ensure data accuracy and relevance, CiteSpace-based deduplication and manual thematic screening were performed to exclude irrelevant entries. Two independent investigators reviewed the titles and abstracts, excluding articles not related to pediatric NS, which resulted in the inclusion of 2,886 publications. Finally, 13 articles published in 2026 were excluded via CiteSpace, resulting in 2,873 eligible publications included in the analysis. The study's retrieval and selection process are illustrated in Figure 1. Since the data did not involve any identifiable patient information, this study did not necessitate an ethical review. Journal information, including impact factor (IF), category and Journal Impact Factor (JIF) quartile (Q1–Q4), was collected from the 2024 Journal Citation Reports.

### 2.3 Analysis tool

Statistical analyses were primarily conducted using CiteSpace (Version 6.3. R3) (5) and VOSviewer (Version 1.6.20) (6). Additionally, the Bibliometrix package in R (Version 4.5.2) was used for statistical analysis and visualization of publication trends, types, and distributions. CiteSpace settings included a 1-year “time slice” value, selection of different network clipping methods based on graphical complexity, the G-index factor K set to 25, and TopN% set at 10%. The configuration of image nodes and connections was tailored to the specific objective of each analysis. VOSviewer analysis and R was conducted using the default settings.

## 3 Results

### 3.1 Annual publication trends

**Figure 2:**
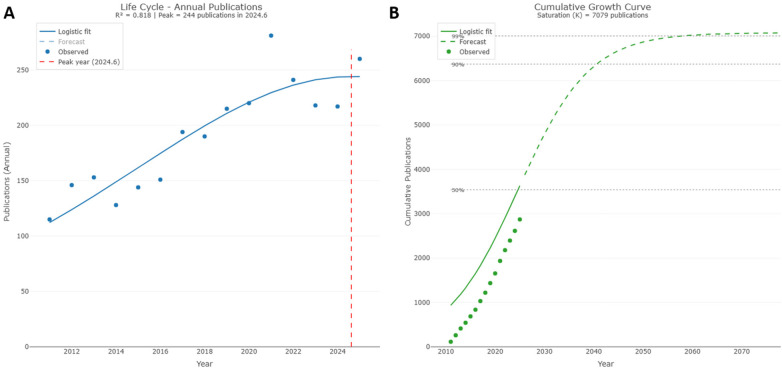
Life cycle analysis of scientific production in the field of pediatric NS. **(A)** Logistic life cycle curve of annual publications. **(B)** Logistic growth curve of cumulative publications.

A total of 2,873 documents were included in this study, comprising 2,688 articles and 185 review articles. Two line charts (Figure 2) were generated using R to depict the publication trends of research on pediatric NS over the 15 years, illustrating the developmental progression of research on it. In picture A, the *x*-axis represents the year, and the *y*-axis indicates the annual number of publications. From 2011 to 2025, the annual number of publications exhibited a steady yet fluctuating upward trend. However, a notable surge occurred in 2017 (publications = 194), with the annual output reaching a significant high before climbing to a peak publications in 2024. In picture B, the *x*-axis also represents the year, while the *y*-axis denotes the cumulative publications. This presents the cumulative growth trend fitted by a logistic model, with a theoretical saturation (K) of 7,079 publications. The total cumulative number of included publications was 2,873, accounting for approximately 40.58% of the K value, indicating that the research field is still in the rapid growth stage and has not yet reached the mature saturation phase (7).

### 3.2 Contribution of countries

During the past 15 years, 111 countries have successively conducted research on the field of pediatric NS, among which China ranked first globally in output with 452 publications, followed by the India and United States with 367 and 352 papers, respectively (Table 1). Notably, although China held the highest publication volume, its betweenness centrality was comparatively low (Centrality = 0.01). This disparity indicates that while China produces significant research, its function as a structural bridge linking various national research clusters is less pronounced than that of European and American countries such as France (0.15) and Canada (0.10), which serve as pivotal hubs in the global collaborative network.

**Table 1:** Top 10 countries in terms of publications.

Rank	Country	Publications	Centrality
1	China	452	0.01
2	India	367	0.07
3	United States	352	0.08
4	Japan	265	0.00
5	Italy	156	0.03
6	Türkiye	154	0.02
7	UK	130	0.09
8	Canada	130	0.10
9	France	121	0.15
10	Egypt	120	0.09

**Figure 3:**
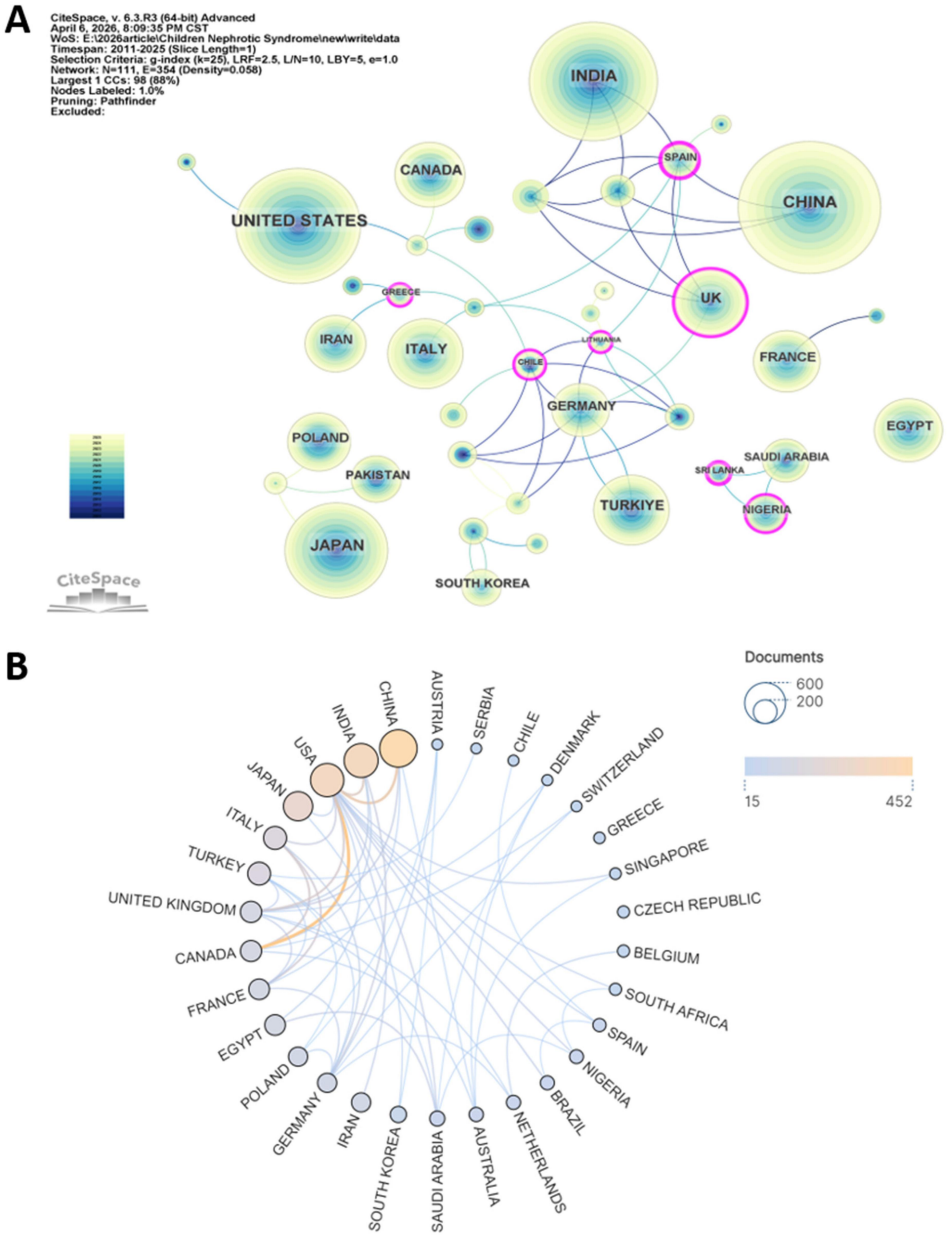
**(A)** The network map of cooperation between counries. This network visualization was generated using CiteSpace 6.3.R3 to illustrate international academic collaborations in pediatric NS research from 2011 to 2025. Nodes represent individual countries, with its size proportional to the number of publications of each country. Lines between countries indicate collaborative relationships. Nodes highlighted in purple denote countries with high centrality. **(B)** The radial diagram of cooperation between counries. The radial diagram was generated by VOSviewer and Scimago Graphica, which illustrates cross-country collaborative ties in pediatric NS research from 2011 to 2025. Node size indicating the total number of publications. The color of nodes corresponds to publication volume, ranging from 15 to 452 documents.

Figure 3A presents the global country collaboration network visualized via CiteSpace. The network comprises 111 countries and 354 collaborative ties. Node size directly reflects the volume of publications contributed by each country, larger nodes represent better research output. The purple outer rings indicate elevated betweenness centrality values. Figure 3B presents a radial diagram generated using VOSviewer and Scimago Graphica, illustrating the international research collaboration landscape among 30 countries in the field of pediatric NS. In this circular country co-authorship network, node size indicating the total number of publications. The color of nodes further corresponds to publication volume, ranging from 15 to 452 documents. The regional collaboration landscape shows clear clustered patterns. The North American-European core, comprising the United States, Canada, the United Kingdom, and France, forms a high intensity collaborative cluster. This structural prominence is consistent with their high betweenness centrality, as reflected in the scores of 0.15 for the United Kingdom and 0.10 for France. Meanwhile, the Asian research cluster consisting of China, Japan, and India shows substantial publication volumes. However, their collaborative ties are more limited in scope compared with the North American-European hub. This disparity indicates that these countries have considerable potential to further expand their cross-continental partnerships in the future. Furthermore, secondary hubs such as Italy, Türkiye, and Egypt act as vital nodes that maintain stable collaborative links across European, Asian, and African regions.

### 3.3 Institution analysis

To maintain the integrity and consistency of the collaboration network, university systems were consolidated with their directly affiliated clinical centers into unified nodes. Following this normalization process, 385 institutions were identified as having contributed to research on pediatric NS, with the ten most prolific institutions detailed in Table 2. Assistance Publique Hopitaux Paris (publications = 77, centrality = 0.02) tops the list as the most prolific institution, followed by Kobe University (69, 0.20) and Universite Paris Cite (66, 0.00). France exhibits a formidable presence and significant strategic emphasis in pediatric NS, with three institutions, including Assistance Publique Hopitaux Paris, Universite Paris Cite, and Institut National de la Sante et de la Recherche Medicale, ranking among the top ten.

**Table 2:** Top 10 institutions in terms of publications.

Rank	Institution	Country	Publications	Centrality
1	Assistance Publique Hopitaux Paris	France	77	0.02
2	Kobe University	Japan	69	0.20
3	Universite Paris Cite	France	66	0.00
4	National Center for Child Health and Development	Japan	58	0.02
5	All India Institute of Medical Sciences New Delhi	India	53	0.04
6	University of Toronto	Canada	52	0.20
7	University System of Ohio	United States	50	0.03
8	University of London	United Kingdom	50	0.02
9	Institut National de la Sante et de la Recherche Medicale	France	49	0.06
10	IRCCS Bambino Gesu	Italy	48	0.13

**Figure 4:**
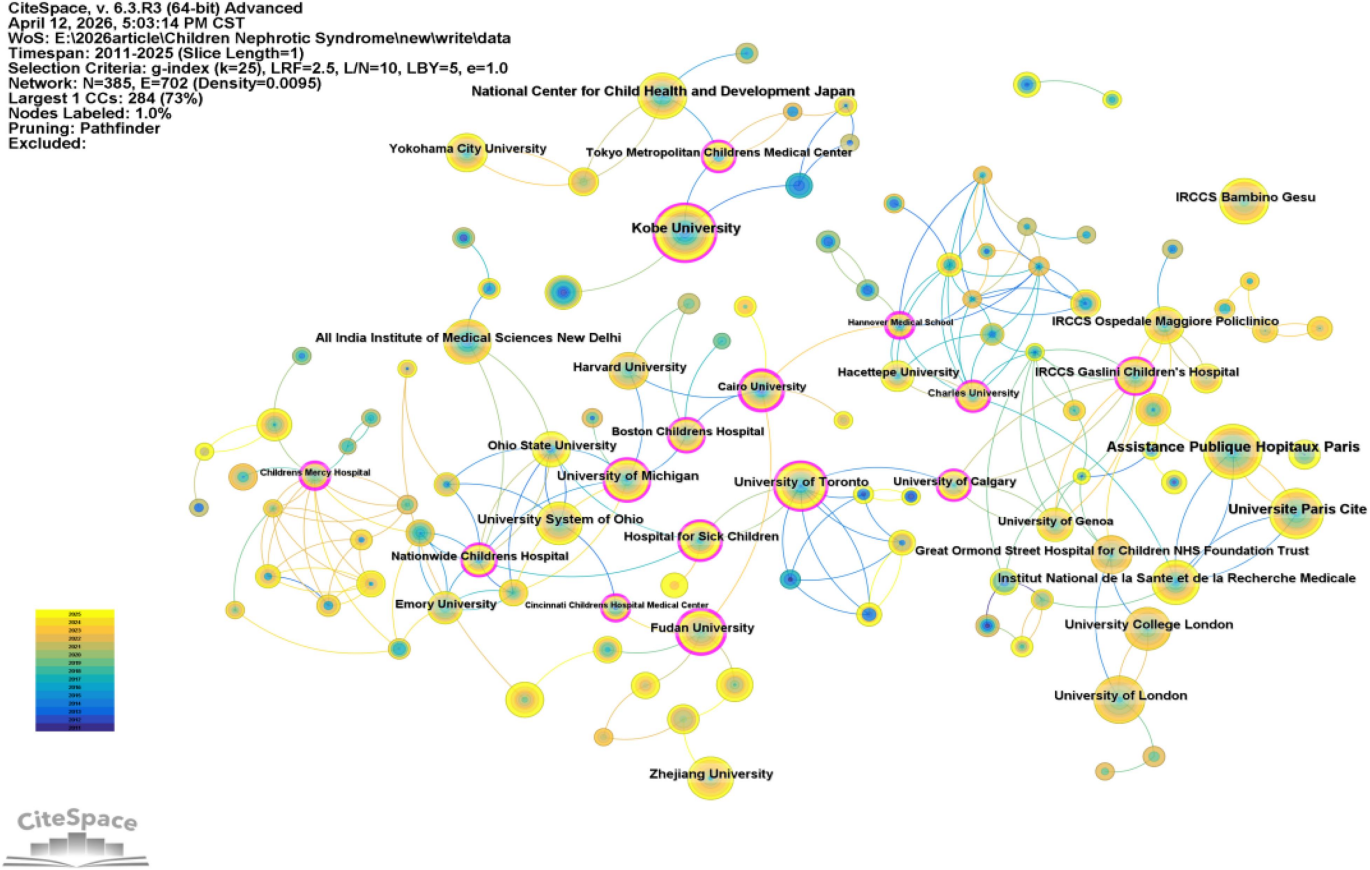
The co-occurrence network map of institutions. The co-occurrence network map of institutions. This network visualization was generated using CiteSpace 6.3.R3 to illustrate academic collaborations among institutions in pediatric NS research from 2011 to 2025. Nodes represent individual institutions, with node size proportional to their publication output. Lines between nodes indicate collaborative relationships. Nodes highlighted in purple denote institutions with high centrality.

An institutional co-occurrence map was generated with institutions depicted as nodes (Figure 4). Analysis of the network reveals that Cairo University in Egypt (25, 0.34) functions as a core entity within the inter-institutional cooperation framework. The network density stands at 0.0095, signifying a relatively low level of coordination among institutions researching pediatric NS globally. These findings suggest that enhancing cross-institutional synergy and leveraging the unique resources of diverse academic hubs would be instrumental in advancing the clinical understanding and treatment of this condition.

### 3.4 Author analysis

A total of 615 authors have contributed to the field of pediatric NS. The ten most productive authors are listed in Table 3. Iijima Kazumoto is the most prolific author with 58 publications, followed by Nozu, Kandai (publications = 45) and Kamei Koichi (43), respectively. Notably, Boyer Olivia is not only one of the most productive authors, but she also has the highest betweenness centrality (centrality = 0.20) in the co-authorship network. This simultaneous prominence highlights her critical function as a principal academic conduit and a foremost authority in the international pediatric NS research community.

**Table 3:** Top 10 most productive authors.

Rank	Authors	Publications	Centrality
1	Iijima, Kazumoto	58	0.01
2	Nozu, Kandai	45	0.07
3	Kamei, Koichi	43	0.05
4	Vivarelli, Marina	42	0.03
5	Ishikura, Kenji	42	0.04
6	Bagga, Arvind	38	0.07
7	Boyer, Olivia	36	0.20
8	Ito, Shuichi	34	0.05
9	Emma, Francesco	30	0.02
10	Mao, Jianhua	29	0.06

**Figure 5:**
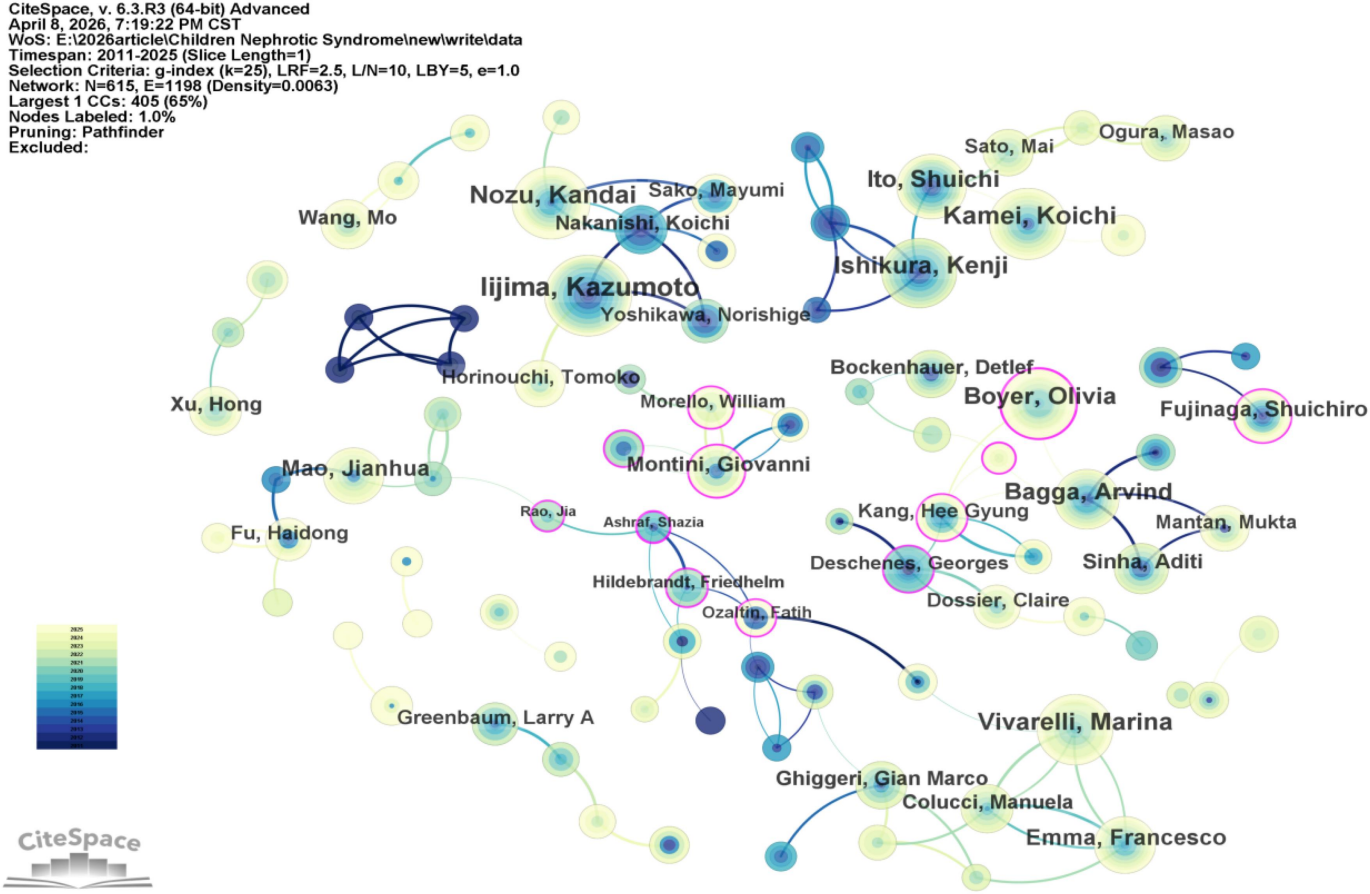
Collaboration network of authors. This network visualization was generated using CiteSpace 6.3.R3 to illustrate collaborative relationships among authors in pediatric NS research from 2011 to 2025. Nodes represent individual authors, with node size proportional to their publications. Lines between nodes indicate collaborative ties. Nodes highlighted in purple denote authors with high centrality.

A collaboration network graph of authors was produced by CiteSpace (Figure 5). Price's law stipulates that the minimum number of publications necessary for core authors is *n* = 0.749 $\sqrt{Nmax}$, where Nmax represents the publication count of the most productive author (7). Consequently, the minimum number of publications required for an author to be considered a core author in the field of pediatric NS research rounds to 6 articles (5.704). There are 104 core authors who have collectively published a total of 1,411 papers, which constitutes 51.29% of all articles. According to Lotka's law, core authors should make up 50% of all published works (8). In this analysis, the percentage of publications by core writers was 51.29%, which is close to the theoretical value. This shows that a stable group of core authors has evolved in the pediatric NS area.

### 3.5 Analysis of journals

All the searched literatures came from 830 journals. Table 4 presents results derived from the Bibliometrix package in R, Which enumerates journals based on research output and presents essential indicators such as IF, category, JIF quartile, and H-index. Pediatric Nephrology ranks first with 385 publications and the highest H-index of 38, succeeded by Frontiers in pediatrics (Output = 88) and Clinical and Experimental Nephrology (57). The highest IF is held by Kidney International Reports (IF = 5.7). Among the 10 journals included, half are ranked Q1/Q2, and the other half are ranked Q3/Q4. Higher H-index values indicate a lasting citation impact. This holds true even for journals with a moderate IF. For instance, Pediatric Nephrology has the highest H-index (38) and the most outputs (338), despite having an IF of only 2.6. Conversely, the presence of a greater number of publications does not necessarily equate to improved rankings or higher impact factors. For example, Saudi Journal of Kidney Diseases and Transplantation has a relatively high publication output (52). However, it has a comparatively low H-index of 11 and an impact factor of 0.3. In contrast, Nephrology Dialysis Transplantation shows a significantly higher academic impact, with an H-index of 21 and an IF of 5.6, despite its lower publication output (34).

**Table 4:** Top 10 most productive journals.

Rank	Journal	Output	IF	Category	JIF	H-index
1	Pediatric Nephrology	385	2.6	Pediatrics/Urology and Nephrology	Q1/Q2	38
2	Frontiers in Pediatrics	88	2.0	Pediatrics	Q2	16
3	Clinical and Experimental Nephrology	57	1.7	Urology and Nephrology	Q3	17
4	Saudi Journal of Kidney Diseases and Transplantation	52	0.3	Urology and Nephrology	Q4	11
5	BMC Nephrology	49	2.4	Urology and Nephrology	Q2	10
6	Indian Journal of Nephrology	48	0.8	Urology and Nephrology	Q4	10
7	Iranian Journal of Kidney Diseases	37	0.7	Urology and Nephrology	Q4	9
8	Nephrology Dialysis Transplantation	34	5.6	Transplantation/Urology and Nephrology	Q1/Q1	21
9	Kidney International Reports	30	5.7	Urology and Nephrology	Q1	11
10	Pediatrics International	30	0.9	Pediatrics	Q3	9

**Figure 6:**
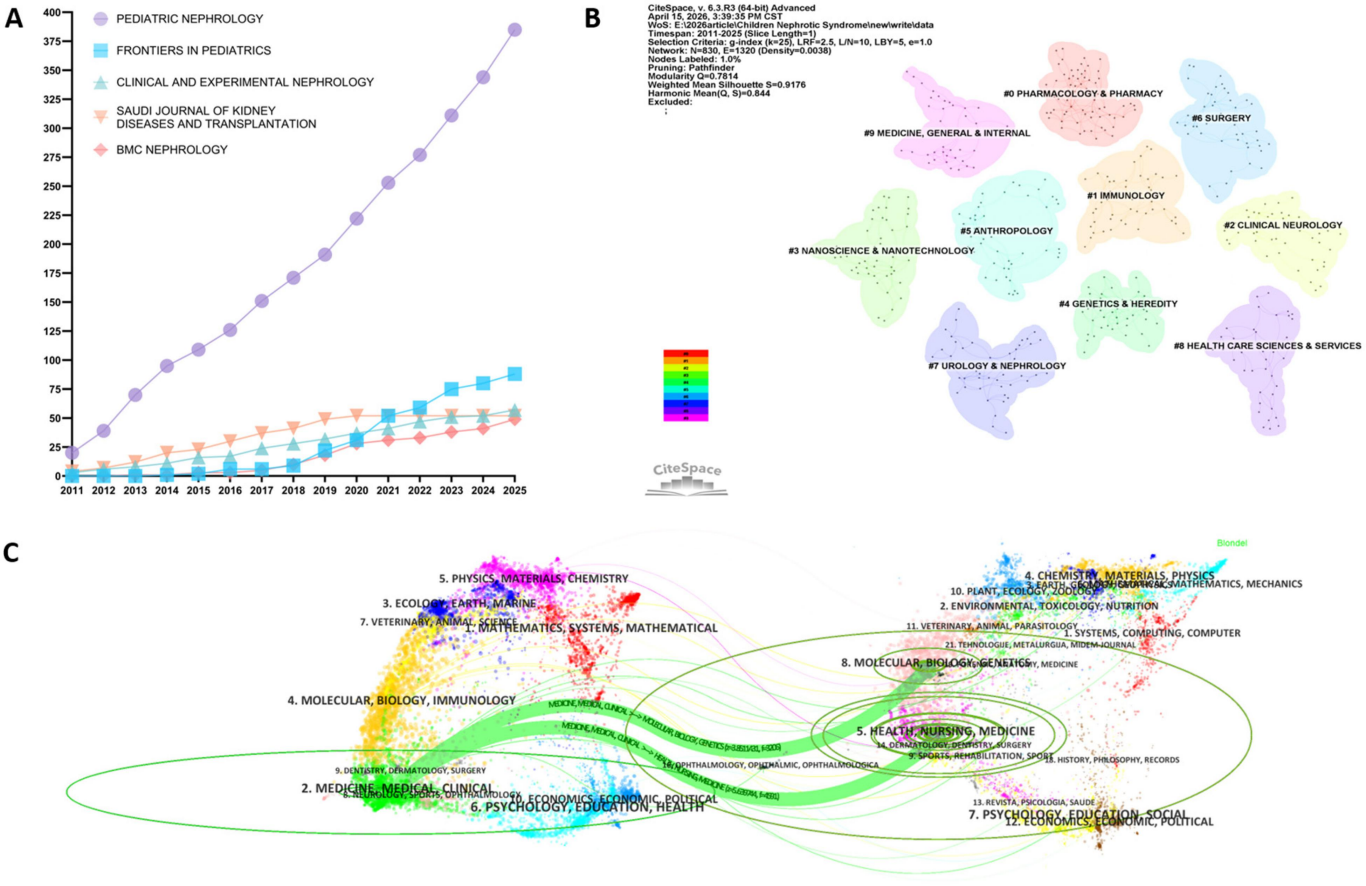
**(A)** TOP 5 journals production over time. This line chart illustrates the cumulative publication output of the top 5 most productive journals in pediatric NS research from 2011 to 2025. The *y*-axis represents cumulative publication counts, while the *x*-axis spans the study year. Among the five journals, Pediatric Nephrology exhibits the most pronounced growth, with cumulative publications 385 by 2025, establishing it as the dominant core venue for this field. **(B)** Co-citation analysis of journals. This visualization was generated using CiteSpace 6.3.R3 to illustrate co-citation relationships among journals in pediatric NS research from 2011 to 2025. **(C)** The dual-map overlay of journals. This dual-map overlay was generated using CiteSpace 6.3.R3 to visualize the disciplinary distribution and citation flows of journals in pediatric NS research from 2011 to 2025. The coloured pathway denotes citation relationships, featuring citing journals on the left and cited journals on the right. The green arcs represent the principal citation pathway.

Figure 6A illustrates the cumulative publication output of the top 5 most productive journals visualized by year. Pediatric Nephrology exhibits a distinct growth trend, with its cumulative output surging from 20 occurrences in 2011 to 385 by 2025, emerging as the most productive journal overall. The plot highlights the rapid expansion of pediatric NS research output, driven primarily by Pediatric Nephrology, while other core journals show sustained but more gradual publication growth.

Co-citation frequency is a critical metric for evaluating the scientific impact of a journal. The journals are arranged in Table 5 according to their incidence of co-citation. Pediatric Nephrology leads with 2,242 citations. Next is Kidney International at 1,545 citations. Journal of the American Society of Nephrology follows with 1,390 citations. Lancet has the highest IF at 88.5. Meanwhile, Pediatric Nephrology boasts the top H-index of 38. Most journals are classified as Q1. Figure 6B visualizes the journal co-citation network, emphasizing the frequently co-cited journals and illustrating their relationships and clustered disciplinary structure.

**Table 5:** Top 10 most co-citation in journals.

Rank	Journal	Citation	IF	Category	Quartiles	H-index	Rank
1	Pediatric Nephrology	2,242	2.6	Pediatrics/Urology and Nephrology	Q1/Q2	38	1
2	Kidney International	1,545	12.6	Urology and Nephrology	Q1	19	2
3	Journal of the American Society of Nephrology	1,390	9.4	Urology and Nephrology	Q1	21	3
4	Nephrology Dialysis Transplantation	1,215	5.6	Transplantation/Urology and Nephrology	Q1/Q1	21	4
5	The Lancet	1,157	88.5	Medicine, General and Internal	Q1	3	5
6	Clinical Journal of the American Society of Nephrology	1,148	7.1	Urology and Nephrology	Q1	22	6
7	American Journal of Kidney Diseases	934	8.8	Urology and Nephrology	Q1	8	7
8	Pediatrics	730	6.4	Pediatrics	Q1	7	8
9	New England Journal of Medicine	707	78.5	Medicine, General and Internal	Q1	2	9
10	Journal of Pediatrics	704	3.5	Pediatrics	Q1	3	10

Additionally, we generated a dual-map overlay utilising CiteSpace to illustrate the topic distribution of academic articles (Figure 6C). The coloured pathway denotes citation relationships, featuring citing journals on the left and cited journals on the right. The green arcs represent the principal citation pathway. The majority of studies concentrated on “medicine, medical, and clinical.” The referenced publications predominantly pertained to “molecular, biology, genetics,” and “health, nursing, medicine.”

### 3.6 Keyword analysis

**Figure 7:**
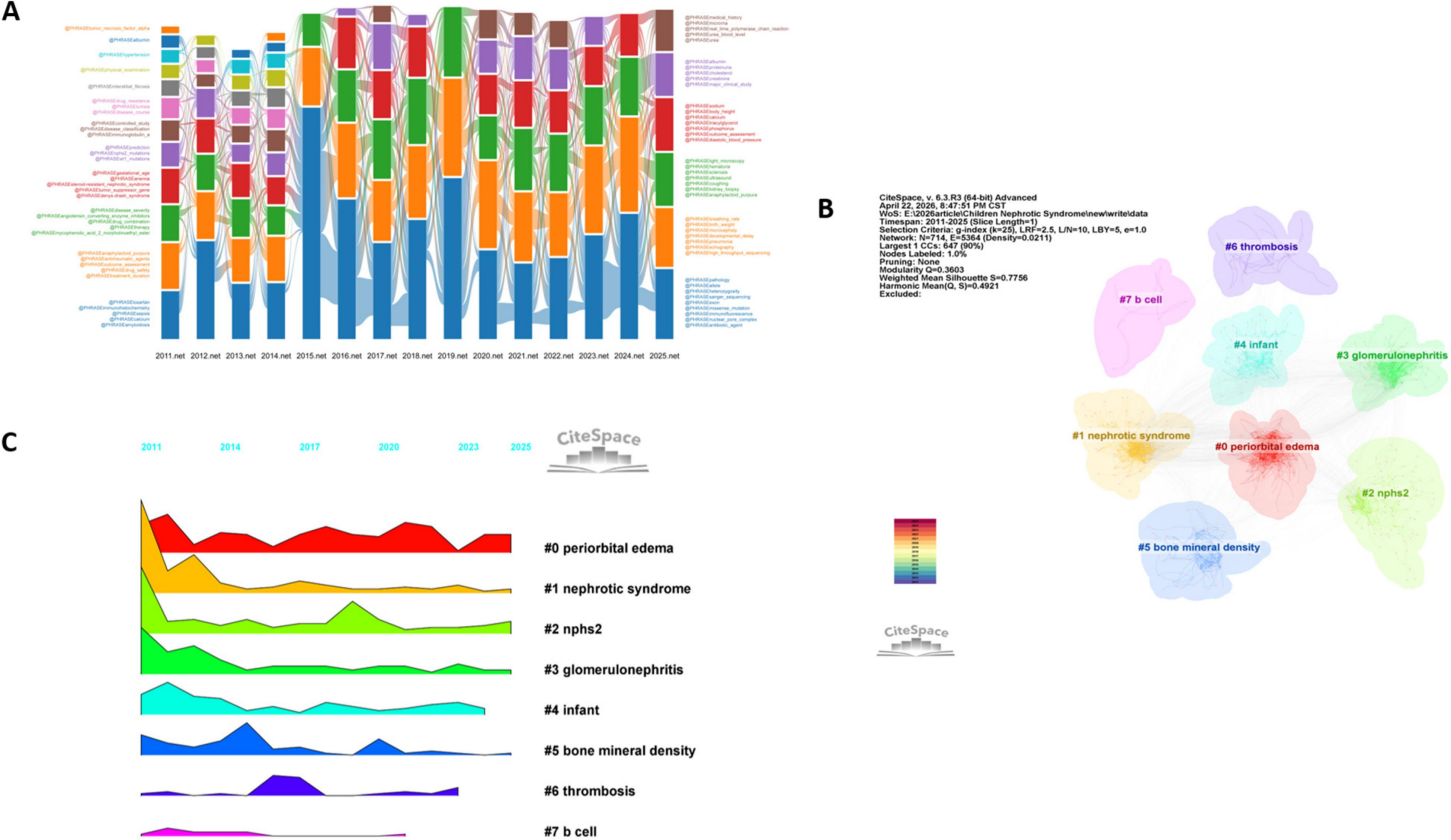
**(A)** alluvial flow map of keywords from 2011 to 2025. This alluvial flow map was generated using CiteSpace 6.3.R3 to visualize the temporal evolution and clustering dynamics of keywords in pediatric NS research from 2011 to 2025. Vertical slices represent annual intervals, while colored horizontal streams denote keywords. The thickness of each stream is proportional to the frequency or intensity of the keyword in publications for that year, and the flow of streams across years reflects the persistence or emergence of research themes over time. **(B)** Knowledge map of keyword clustering. This clustering visualization was generated using CiteSpace 6.3.R3 to illustrate the thematic structure of keywords in pediatric NS research from 2011 to 2025. **(C)** Landscape view of keyword clustering. This landscape visualization was generated using CiteSpace 6.3.R3 to illustrate the temporal evolution of thematic clusters derived from keywords in pediatric NS research from 2011 to 2025. Each horizontal row represents a distinct thematic cluster, and the height of the colored curve within each row reflects the relative research activity or citation intensity of that cluster in a given year.

Keyword analysis maps research landscapes and trends. Figure 7A illustrates the evolution of keywords over time via citespace. In 2011, “losartan”, “steroid-resistant_nephrotic_syndrome”, and “angiotensin_converting_enzyme_inhibitors” were among the most frequently used keywords, with research focus on drug therapy and disease pathogenesis. As time progressed to 2025, the predominant keywords shifted to “high_throughput_sequencing”, “kidney_biopsy”, “proteinuria”, “outcome_assessment”, and “developmental_delay”, with research focus shifting toward clinical practice and precision medicine.

Table 6 lists the ten most frequently occurring keywords, after noise terms were excluded, with two of them being part of the search terms used, indicating a high degree of homogeneity in the literature related to pediatric NS. High-frequency keywords predominantly focus on clinical management and pathogenesis. The most common keywords included “Nephrotic Syndrome” (frequency = 1815), “Children” (1,465), and “Focal Segmental Glomerulosclerosis” (466).

**Table 6:** Top 10 keywords with the highest frequency.

Rank	Frequency	Keywords
1	1,815	Nephrotic Syndrome
2	1,465	Children
3	466	Focal Segmental Glomerulosclerosis
4	397	Proteinuria
5	327	Cyclosporine
6	291	Kidney Biopsy
7	259	Treatment Outcome
8	255	Risk Factor
9	243	Chronic Kidney Disease
10	243	Gene Mutation

CiteSpace cluster maps and landscapes (Figures 7B,C) visualize evolving research hotspots. The keyword clustering knowledge map identifies the 8 major clusters within the field, with a modularity Q of 0.787 and a mean silhouette value S of 0.8447. The values demonstrate that the keywords possess a varied array of features, and the clustering structure is both substantial and significant, facilitating the discovery of research hotspots. The primary labels for our keyword clusters include “periorbital edema”, “nephrotic syndrome”, “nphs2”, “glomerulonephritis”, “infant”, “bone mineral density”, “thrombosis”, and “b cell”.

**Figure 8:**
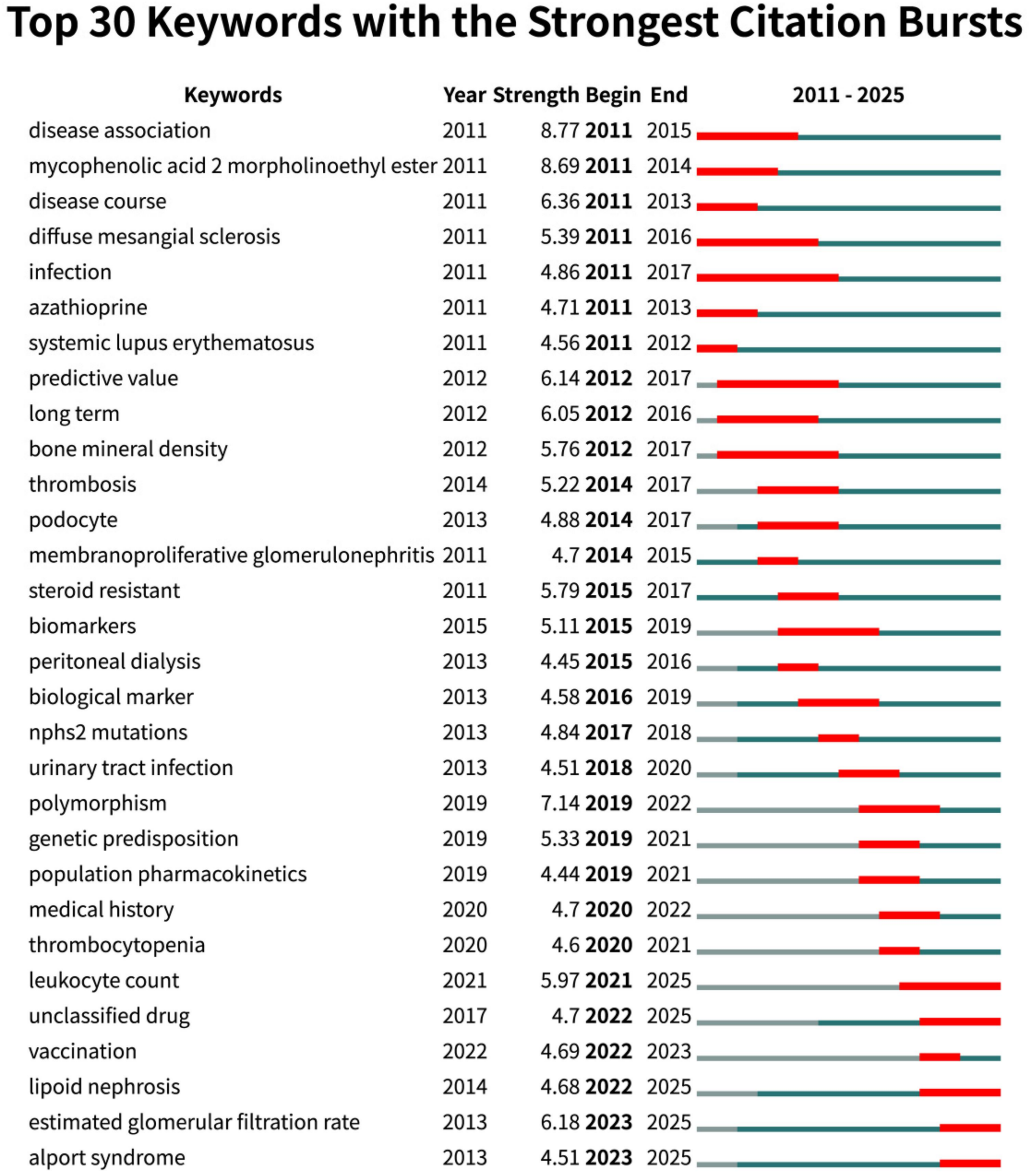
30 keywords in the burst stage. This visualization was generated using CiteSpace 6.3.R3 to analyze citation bursts of keywords in pediatric NS research from 2011 to 2025. The table lists the top 30 keywords ranked by burst strength, with red horizontal bars indicating the temporal duration of each keyword's citation burst.

We analyzed the 30 most significant keywords with strong citation bursts (Figure 8), revealing current research priorities and emerging trends. The red lines represent periods of keyword surges. Burst analysis displayed clear temporal shifts in pediatric NS. This visualization demonstrates that “leukocyte count,” “lipoid nephrosis,” “unclassified drug,” “estimated glomerular filtration rate,” and “alport syndrome” are current research hotspots and potential targets within this field.

### 3.7 Analysis of co-cited references

**Figure 9:**
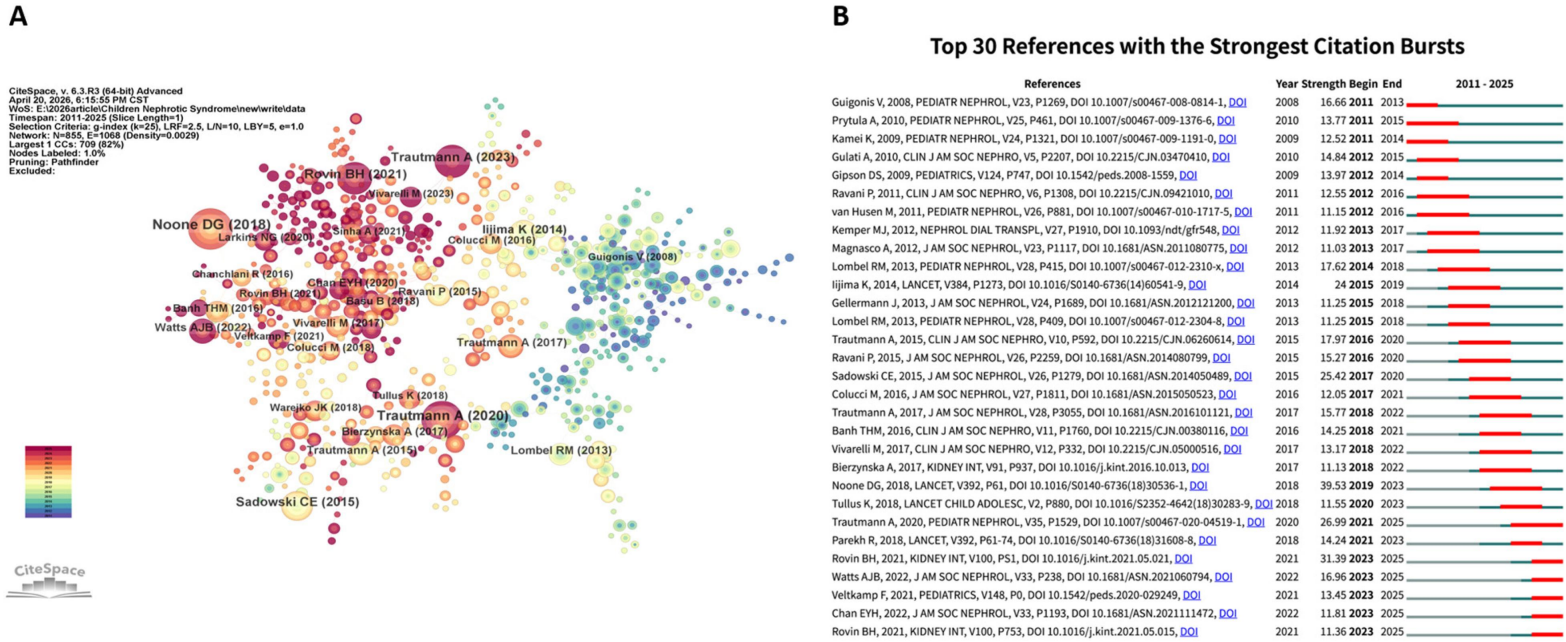
**(A)** Reference co-citation network. This network visualization was generated using CiteSpace 6.3.R3 to illustrate reference co-citation relationships in pediatric NS research from 2011 to 2025. Nodes represent individual cited references, with node size proportional to their co-citation frequency. Lines between nodes indicate co-citation ties between references. Labels mark the first author and publication year of core highly cited references. **(B)** 30 references in the burst stage. This visualization was generated using CiteSpace 6.3.R3 to analyze citation bursts of references in pediatric NS research from 2011 to 2025. The table lists the top 30 references ranked by burst strength, with red horizontal bars indicating the temporal duration of each reference's citation burst.

Figure 9A show the intellectual base and studies of landmark in the research area of pediatric NS with the mashup of reference co-citation analysis.

Citation burst detection clearly depicted the developmental shifts of major research hotspots over time (Figure 9B). The study of Damien G Noone (9) also demonstrates the strongest burst (39.53) starting in 2019, indicating extensive and in-depth explorations have been carried out in the field of childhood idiopathic NS recently. Moreover, ongoing recent burst (2021–2025) for Agnes Trautmann (10) spotlight that the current emphasis in academia is very heavily focused on International Pediatric Nephrology Association clinical practice recommendations for the diagnosis and management of children with steroid-resistant NS.

## 4 Discussion

### 4.1 Growth in publication output and research interest

The bibliometric analysis of 2,873 articles on pediatric NS from 2011 to 2025 reveals a consistent upward trajectory in cumulative publications. We further applied a Logistic growth curve model to quantitatively characterize the field's developmental lifecycle and long-term research potential (Figure 2). While the field maintained steady output in the early 2010s, a foundational shift occurred in 2017, potentially driven by the clinical standardization of biological agents and the increasing accessibility of genomic sequencing for steroid-resistant cases (11). A historical surge in scholarly output occurred in 2021, which was synergistically driven by multiple factors. Specifically, these factors included technological advancements in glomerular laser microdissection combined with mass spectrometry (12), the long-awaited release of the “KDIGO 2021 Clinical Practice Guideline for the Management of Glomerular Diseases” (13), urgent research efforts focusing on the association between SARS-CoV−2 infection and glomerular injury (14), as well as Immunosuppressive therapy has gradually matured (15).

### 4.2 Countries, institutions, authors and journals

From a geographical perspective, academic research in this field is mainly concentrated in Asia, North America and Europe, among which China, India and the United States are the most productive countries. While China leads in absolute publication volume (publications = 452), its centrality remains at 0.01. This may indicate that their research is more localised or less integrated into global collaboration networks. In contrast, Western nations such as France and Canada exhibit the greatest centrality ratings (0.15 and 0.10, respectively), although having less articles (121 and 130, respectively). This suggests that international collaboration and citation impact are more pronounced in the West, probably owing to significant financial resources, sophisticated research facilities, and extensive worldwide collaboration networks, which systematically emphasise cross-border data sharing rather than individual output quantity (16). Moreover, disparities in academic assessment criteria may promote extensive independent research in Asia, whereas the rigorous regulatory framework concerning medical data sovereignty may constrain the integrative potential of high-output countries, thereby perpetuating a decentralised research paradigm despite their substantial scholarly contributions (17). India, as the second most productive country in global pediatric NS research, owes to its large population base and the high regional incidence of NS (9−10 cases per 100,000 children), which is notably higher than that in Western countries (18). The scarce of low-income countries from the top ten list does not diminish the severity of pediatric NS in these regions. Instead, it underscores systemic challenges such as economic constraints and limited research infrastructure. Glomerular diseases constitute a substantial portion of chronic kidney disease in low and middle income countries, yet the epidemiological profile remains largely uncharted due to restricted access to nephropathology services and safety concerns regarding renal biopsies (19). Efforts to harmonize multi-center clinical protocols, such as international biopsy registries and collaborative treatment consortia, are critical to avoid reinforcing health inequities through localized clinical biases.

French institutions are heavily involved in pediatric NS research, with three of them ranking among the top 10 countries in terms of publication volume. Iijima Kazumoto, from Kobe University Graduate School of Medicine, is the most prolific author, focusing on pediatric NS research, with his latest work being “Early detection of proteinuria to prevent kidney fibrosis and progression to kidney failure: lessons from the School Urine Screening Program in Japan” published in Pediatric Nephrology in 2025. This review emphasizes the necessity of reevaluating the role of proteinuria screening in pediatric nephrology and promotes evidence-informed policy decisions that acknowledge its long-term benefits (20). According to Price's law, the core author group within the pediatric NS domain has formed.

The leading ten journals for publications on pediatric NS primarily fall within the fields of Urology and Nephrology and are of superior quality. Among them, Pediatric Nephrology is the flagship journal of the field. It maintains an overwhelming publication advantage, with its total output exceeding that of the other four leading journals combined. Journal co-citation analysis confirms that pediatric NS research is rooted in Urology and Nephrology, closely connected with immunology, genetics, and pharmacology. These disciplinary links are key to the field's ongoing growth. Journals publishing articles on pediatric NS mainly span “medicine, medical, and clinical fields,” while the most cited journals predominantly cover “molecular, biology, genetics” and “health, nursing, medicine”.

### 4.3 Trends in pediatric NS research

The integration of co-citation networks, keyword co-occurrence analysis, and burst detection offers a multidimensional perspective on the intellectual evolution of pediatric NS research over the past 15 years. Temporal shifts in research priorities emerge distinctly through co-citation bursts and keyword dynamics. A keyword analysis conducted by CiteSpace from 2011 to 2025 reveals “nephrotic syndrome,” “children,” and “focal segmental glomerulosclerosis” as the most prominent keywords.

Clusters including “nphs2,” “bone mineral density,” “glomerulonephritis,” and “periorbital edema” emerge as current popular topics. The fact that NPHS2 can appear as a clustering term suggests that the exploration of the molecular mechanisms of NS has been increasingly valued in recent years. Advances in gene delivery and genome editing offer immense potential for targeted kidney therapies. A review by Tavakolidakhrabadi (21) indicates that NPHS1 mutations can lead to congenital NS with podocyte dysregulation, serving as an excellent model for AAV- or mRNA-based gene replacement. Conversely, mutations in genes such as NPHS2, ACTN4, TRPC6, INF2, COQ2–8, and CRB2 predominantly cause focal segmental glomerulosclerosis or focal segmental glomerulosclerosis-like lesions, for which gene- or protein-replacement therapies hold significant promise. Notably, the cluster “bone mineral density” highlights the particular concern for bone health in pediatric patients with NS. Glucocorticoids are commonly used to treat pediatric NS (22). However, long-term glucocorticoid administration inhibits bone mineralization and negatively impacts the fundamental cellular mechanisms critical for the development and maintenance of bone strength. Steroid use can lead to osteoporosis in children, causing a decline in both bone mineral content and bone mineral density (23). Furthermore, the clustering label “glomerulonephritis” suggests that pediatric NS is intrinsically linked to underlying glomerular inflammation and immune dysregulation. Nephrotic proteinuria is the hallmark of various glomerulonephritides driven by diverse pathogenetic mechanisms, including autoimmune, degenerative, and inflammatory processes (24). Among these, Minimal Change Nephropathy and Focal Segmental Glomerulosclerosis are the most prevalent, predominantly affecting children and young adults and accounting for over 90% of NS cases in individuals under 24 years of age. Despite their high clinical incidence, the exact pathogenesis of minimal change nephropathy and focal segmental glomerulosclerosis remains uncertain. Edema is one of the cardinal clinical features of NS. Periorbital swelling in children with NS is a recurring symptom, either with or without generalized edema. The emergence of the clustering keyword “periorbital edema” suggests a growing emphasis on edema management in recent years (25–27).

Trend topics demonstrate the dynamic evolution of research hotspots in the field of pediatric NS. While early studies between 2011 and 2016 focused on foundational renal histopathology, traditional immunosuppressants and disease course predictors, a transitional phase from 2017 to 2021 shifted toward genetic predisposition, steroid resistance mechanisms and evidence-based guideline development. Current research from 2022 to 2025 is characterized by biologic therapy breakthroughs, genotype-guided diagnosis and long-term renal prognostic management, as evidenced by emerging interest in unclassified drug, genetic variability, estimated glomerular filtration rate, leukocyte count and body mass. This evolution reflects the broader movement of pediatric NS from reactive symptomatic treatment to a proactive, precision-based paradigm.

Burst keyword analysis shows that pathogenesis and prognosis are the main current research hotspots. Vaccination was only briefly active from 2022 to 2023 and have since exited mainstream research. These sustained burst keywords can be categorized into three interconnected research dimensions that define the current landscape.

First, in the domain of disease subtypes and pathogenesis research. “leukocyte count” (burst strength = 5.97), bursting in 2021, underscores the central role of infection induced inflammation in NS. Particularly after the COVID-19 pandemic, kidney involvement in children exhibited various, but mostly mild manifestations (28). Self-limiting proteinuria is a common finding during the COVID-19 infection, but the risk of clinically relevant relapses appears to be low (29). In addition, “lipoid nephrosis” and “alport syndrome” initiated their sustained burst periods in 2022 and 2023 respectively, underscoring the increasing emphasis on precise stratification of NS subtypes. Lipoid nephrosis is also known as minimal change disease. In recent years, studies have focused on the diagnostic value of circulating anti-nephrin antibody in patients with minimal change disease. Chen et al. (30) reported that patients with positive anti-nephrin antibody had lower serum albumin and IgG levels, higher serum lipid levels, and more severe proteinuria. When patients respond to treatment, the anti-nephrin antibody may turn negative. Along with minimal change disease, Alport syndrome has become a hot topic in the study of rare diseases. Alport syndrome is a genetically and phenotypically heterogeneous disease affecting glomerular, cochlear, and ocular basement membranes, which is caused by gene mutations (31). Recent case studies have indicated that NS is the primary clinical presentation in pediatric patients with Alport syndrome (32, 33). This discovery indicates that Alport syndrome should be included in the differential diagnosis of pediatric NS, particularly in instances with unusual clinical manifestations or inadequate response to standard steroid treatment.

Second, in the domain of diagnostic and therapeutic technologies. “unclassified drug” (4.70), which emerged in 2022, refers specifically to B-cell depleting agents including rituximab, ofatumumab and belimumab. The 2021 KDIGO glomerulonephritis guideline update, which upgraded rituximab from a third-line agent in 2012 to a standard treatment option for steroid-dependent pediatric NS, was the direct trigger for the 2022 research burst of “unclassified drug” (13). Patient outcomes have changed dramatically with advancements in immunosuppressants (34). Children with NS exhibit many changes in their immunological homeostasis involving memory B cells. All circulating B-cell subsets are depleted by rituximab, and its therapeutic effectiveness emphasizes the critical role that B cells play in the pathophysiology of NS. Studies indicate that the disease seldom relapses until B cells are fully reconstituted, and restoration of class-switched memory B cell populations is predictive of relapse (35, 36). Furthermore, rituximab therapy successfully corrects the imbalance between effector and regulatory T cells in NS (35, 37). In children with steroid-resistant, CNI-refractory NS, higher response rates to rituximab were observed in those with minimal change disease, late steroid resistance, early rituximab administration, and concurrent immunosuppression (15). Overall, it seems that rituximab is generally safe for the majority of children (11, 38). Recent studies have primarily concentrated on assessing the long-term efficacy and safety of B cell-depleting agents (39, 40), along with the management of side effects, including rituximab-associated hypogammaglobulinaemia (41, 42). In the future, next-generation anti-CD20 antibodies, plasma cell-targeted drugs and cellular therapies will bring new hope for patients with refractory NS (43).

Third, in the domain of prognosis and outcomes. “estimated glomerular filtration rate”, which had the highest burst strength of 6.18 starting in 2023, reflects the field shift from focusing on short-term remission to prioritizing lifelong renal health. This shift is particularly critical in pediatric NS. A study (44) highlights that estimated glomerular filtration rate trajectories in pediatric patients are often significantly “nonlinear” due to frequent relapses and the high heterogeneity of steroid-resistant NS, rendering traditional short-term assessments prone to bias. A 2-year follow-up study (45) demonstrated the clinical value of long-term monitoring. Its results, tracking estimated glomerular filtration rate and albumin-to-creatinine ratio over time, revealed a gradual and consistent improvement in renal function alongside a significant reduction in proteinuria. Notably, these data underscore the efficacy of rituximab therapy in sustaining remission for both steroid-sensitive NS and steroid-resistant NS patients, particularly in the steroid-sensitive NS cohort.

Meanwhile, the most important recent development, marked by Damien G Noone's widely cited paper (strength = 39.53), indicating extensive explorations have been carried out in the field of childhood idiopathic NS from 2019. Moreover, ongoing recent burst for Agnes Trautmann and Brad H. Rovin spotlight that the current emphasis in academia is very heavily focused on clinical practice recommendations for the diagnosis and management of pediatric NS. Collectively, research on pediatric NS diseases of different pathological types remains a key focus, and various monoclonal antibodies (especially rituximab and cyclosporine) are still the main therapeutic approaches under current investigation, showing promising outcomes in both preclinical and clinical studies.

Podocytes are specialised epithelial cells forming the glomerular filtration barrier that regulates the filtration process. NS manifests as excessive protein in the urine, a condition typically caused by podocyte dysfunction (46–48). Nephrin is a key molecule in maintaining the integrity of the glomerular filtration barrier, and its damage constitutes a central pathological mechanism leading to proteinuria (49). Anti-Nephrin Antibody is a class of autoantibodies directed against Nephrin, a key structural protein on the surface of glomerular podocytes. Research indicates that this antibody is present in some patients with minimal change disease and recurrent FSGS, suggesting it represents an autoimmune form of podocytopathy (50). Given the well-defined aetiology of autoimmune conditions, B-cell-targeting agents such as Rituximab demonstrate marked efficacy in reducing antibody levels. Clinical observations indicate that diminishing antibody concentrations frequently coincide with the resolution of proteinuria, suggesting antibody titres may serve as a monitoring parameter for therapeutic response assessment (51). Two recent studies supporting that measuring anti-nephrin antibodies can help predict disease course and treatment response in children (52, 53). In the future, the identification of anti-nephrin antibodies as a pathogenic factor in a subset of primary podocytopathy cases will facilitate a novel, mechanism-based understanding and refined classification of pediatric antibody-mediated podocytopathies (54).

### 4.4 Challenges in pediatric NS

Despite the growing body of research on pediatric NS, several critical clinical issues remain unresolved, as reflected in the current literature. First, despite the favorable efficacy of rituximab in pediatric NS, one important caveat in interpreting the safety profile of rituximab is that most available data come from children older than 6 years of age with a follow-up duration of less than 5 years (15). Consequently, the long-term safety and potential late-onset adverse effects in younger children remain largely unknown. Further prospective, large-scale, multicenter trials with extended follow-up periods are urgently needed. Second, although genetic mechanisms are becoming increasingly clear, targeted therapies for these specific genetic mutations remain scarce. A significant unresolved clinical issue is the lack of disease-modifying agents (55). As highlighted in the current literature, the disease course in frequently relapsing or steroid-resistant cases often spans over a decade, and most available immunosuppressive agents fail to alter the long-term disease trajectory, serving primarily as symptomatic control rather than a cure. Third, although extensive research has been conducted on pediatric NS, the underlying proteinuria-inducing factor(s) have remained elusive for decades. While the recent 2022 discovery of anti-nephrin antibodies in childhood NS (56) has fundamentally shifted the perspective of pediatric nephrologists regarding the pathophysiology of this idiopathic disease, critical gaps remain. Specifically, the age-dependent pathogenesis and immune tolerance mechanisms unique to children require further elucidation (57). Fourth, in addition to medication-related side effects, those with frequently relapsing or steroid-resistant NS are susceptible to complications like severe edema, infections, acute renal injury, and thromboembolism. These patients should be co-managed with a subspecialist and require long-term monitoring and follow-up. Although the short-term benefits of rituximab in achieving relapse-free survival are established, the potential for late-onset adverse effects requires further investigation (58). Additionally, the overall financial burden associated with rituximab treatment is a major concern, particularly in developing nations.

### 4.5 Limitations

This bibliometric study has several limitations. Firstly, data were exclusively sourced from the WoSCC/Scopus database, and only articles published in English were considered. This reliance on English-centric database introduces a potential selection bias and may result in overlooking significant non-English and regional publications from Low-and Middle-Income Countries, as well as research embedded within specific cultural contexts. Furthermore, while manual screening was employed to ensure relevance, the process inherently carries the risk of information omission. Thirdly, citation-based analysis tends to favor older, established literature and reflects academic influence rather than practical clinical impact. Finally, bibliometric analysis tools come with intrinsic limitations and biases, which could impact the analysis outcomes.

## 5 Conclusion

This study utilized bibliometric methods and CiteSpace visualization to systematically evaluate the global development of pediatric NS research from 2011 to 2025, tracking a significant increase in scholarly output that reached a historical peak in 2024. Geographically, while the China emerged as the leading contributor with 452 publications, its 0.01 centrality highlights a distinct volume-centrality disparity compared to European nations like France, which maintain the highest structural influence (centrality = 0.15) as primary collaborative hubs. The thematic evolution of the field followed a clear three-stage trajectory: early research (2011–2016) focused on foundational renal histopathology, traditional immunosuppressants and disease course predictors; a transitional middle phase (2017–2021) shifted toward genetic predisposition, steroid resistance mechanisms and evidence-based guideline development; and current trends (2022–2025) are characterized by biologic therapy breakthroughs, genotype-guided diagnosis and long-term renal prognostic management. Despite these advancements, several limitations warrant consideration, including the exclusive reliance on the English-language WoSCC/Scopus database and the potential omission of regional publications from Low-and Middle-Income Countries. Ultimately, bridging the current translational gaps will necessitate enhanced international collaboration，particularly between high-volume Asian contributors and established European steering committees to move the field toward a proactive, data-driven precision paradigm.

Overall, research on pediatric NS holds considerable promise. However, despite the growing body of literature, critical clinical challenges persist. Therapeutically, the long-term safety of rituximab in younger children remains ill-defined, and current immunosuppressants largely offer symptomatic control rather than disease modification, complicating long-term management amidst economic constraints. Mechanistically, while the discovery of anti-nephrin antibodies has reshaped etiological perspectives, the age-dependent pathogenesis and immune tolerance mechanisms unique to children remain to be fully elucidated. This analysis enables researchers to rapidly grasp the current landscape, emerging trends, and frontiers in the field, informs future research directions, and provides a theoretical foundation for the clinical management of pediatric NS.

## Data Availability

The raw data supporting the conclusions of this article will be made available by the authors, without undue reservation.
